# Chlorinated and brominated organic pollutants in shellfish from the Yellow Sea and East China Sea

**DOI:** 10.1007/s11356-014-3198-8

**Published:** 2014-06-25

**Authors:** Ge Yin, Lillemor Asplund, Yanling Qiu, Yihui Zhou, Hua Wang, Zongli Yao, Jianbin Jiang, Åke Bergman

**Affiliations:** 1 Department of Materials and Environmental Chemistry, Stockholm University, 10691 Stockholm, Sweden; 2 College of Environmental Science and Engineering, Tongji University, 200092 Shanghai, China; 3 East China Sea Fisheries Research Institute, Chinese Academy of Fishery Sciences, 200090 Shanghai, China; 4 Tongzhou Fisheries Technical Instruction Station of Nantong, 226300 Nantong, China

**Keywords:** Shellfish, Pesticides, HBCDD, DDT, MeO-PBDE, China

## Abstract

The global contamination with persistent organic pollutants (POPs), or compounds with similar characteristics, is well known. Still there are data gaps for POP concentrations from many areas in the world. The aim of the present study is to assess several legacies POPs and also hexabromocyclododecane (HBCDD) and methoxylated polybrominated diphenyl ethers (MeO-PBDEs) in shellfish from three locations in the Yellow Sea and East China Sea. The sources of the contaminants are discussed. Pooled samples were treated by liquid-liquid extraction and acid and column cleanup prior to analysis by gas chromatogram equipped with electron capture detector (GC-ECD) and gas chromatography-mass spectrometry (GC-MS). The by far most abundant environmental contaminant originates from dichlorodiphenyltrichloroethane (DDT), independent of species analyzed or sampling site. The results indicate ongoing or at least recent discharges of DDT. The second highest concentrations were reported for HBCDD (21–40 ng/g fat) in the shellfish, independent of sampling sites. The two natural products, 6-MeO-BDE-47 and 2′-MeO-BDE-68, were also present in the shellfish (1.3–22 and 1–14 ng/g fat, respectively). The polychlorinated biphenyl (PCB) congener CB-153 (0.8–6.5 ng/g fat), hexachlorobenzene (HCB) (1.1–3.6 ng/g fat), and β-hexachlorocyclohexane (β-HCH) (2.3–4.9 ng/g fat) were all higher than the concentrations of other HCH isomers, β-endosulfan, PBDE congeners, and mirex. Apart from the DDTs and HBCDDs, it is evident that the pollution of shellfish was similar to, or lower than, the contamination of shellfish in other parts of the world.

**Electronic supplementary material:**

The online version of this article (doi:10.1007/s11356-014-3198-8) contains supplementary material, which is available to authorized users.

## Introduction

Persistent organic pollutants (POPs) are lipophilic organic chemicals with low chemical reactivity allowing them to undergo long-range transport, making them bioaccumulative and toxic to wildlife and humans (UNEP 2014). POPs can be classified in three categories according to their sources: pesticides, industrial chemicals, and by-products. Large amount of organochlorine pesticides (OCPs), polychlorinated biphenyls (PCBs), and polybrominated diphenyl ethers (PBDEs) have been manufactured and used in China for several decades.

Being the country with the world’s largest population, China has produced and consumed large amount of pesticides for crop protection and disease vector control (Wong et al. 2005). For instance, 400 thousand tonnes of dichlorodiphenyltrichloroethane (DDT) and 4.9 million tonnes of hexachlorocyclohexanes (HCHs) from the 1950s to 1983 were produced for agricultural use (Fu et al. 2003; Li et al. 1998). Even though China initiated bans of pesticides for certain uses from 1980s and ratified the Stockholm Convention in 2004, some specific exemptions were requested. For example, DDT is still used for malaria control while mirex and chlordane have been used as termiticides (Wong et al. 2005). Pesticides residue level detected in China environment and human are still relatively high even if some data indicated descending temporal trends (Wong et al. 2002). There is, however, a shortage of data from China to make any firm conclusions on general levels spatial and temporal trends of POPs in China.

PCBs have been used for their good electrical insulating properties since the 1930s. However, it was not until 1966 that PCBs were detected in wildlife from the marine environment (Jensen et al. 1969). In 1968, the Yusho incident occurred in Japan (Yoshimura 2003). One thousand and eight hundred people were poisoned by PCB-contaminated rice oil. It was reported that 8,000 tons of PCBs had been manufactured from the 1960s to 1970s (Jiang et al. 1997; Qiu et al. 2012) in China, and technical mixtures named #1 PCB (chlorine content 42 % similar to Aroclor 1242) and #2 PCB (chlorine content 56 % similar to Aroclor 1254) were produced. PCBs were restricted from the 1970s in China. PCB concentrations in environmental media in China are relatively low on a national scale, and it still could be a risk for humans and wildlife due to exposure to PCB-containing equipment and by-product from combustion (Xing et al. 2005).

PBDEs and hexabromocyclododecanes (HBCDDs) are brominated flame retardants used in primarily polymeric materials for improving their fire safety. It was reported that the domestic production demand of brominated flame retardants was 10,000 tonnes in 2000 and has increased by an annual rate of 8 % (Mai et al. 2005). Materials and goods are sources of general PBDE and HBCDD contamination, while e-waste recycling has created severe hot spots.

Methoxylated PBDEs (MeO-PBDEs) are neutral chemicals that seem to primarily originate from natural sources, particularly if the methoxy group is located to the *ortho*-position to the diphenyl ether bridge. They can be also formed from the metabolism of PBDEs (Feng et al. 2010). MeO-PBDEs have been identified in algae, blue mussel, and seals (Haglund et al. 1997; Lofstrand et al. 2011; Malmvarn et al. 2005). It has been suggested as a potential source of hydroxylated PBDEs (Wan et al. 2009).

Shellfish (e.g., mussels and clams) are popular seafood for people living around the coastline of China. Mussels have been widely used as a biomarker for detecting pollutants and monitoring the contamination all over the world, due to their wide distribution, low mobility, and high filtration capacity (Goldberg et al. 1978; O’Connor 2002; Ramu et al. 2007a). They live attached to rocks by its byssus and feed on plankton and other primary producers. Similar to mussels, clams feed on plankton by filter feeding. However, clams are kinds of cave sediment bivalves rather than attaching to rocks.

The Yellow Sea (YS) and East China Sea (ECS) are two of the continental seas of China. The two longest rivers, Yangtze River and Yellow River, are flowing into the ECS and YS, respectively. The coasts of the YS and ECS are densely populated and the most developed in China. The seawater has been used for fishing for a long time. For example, Zhoushan fishing ground, located in ECS, is the biggest fish ground in China. It is famous for croceine croaker, octopus, and cuttlefish. However, the seawater quality in YS and ECS according to national seawater quality standard has become increasingly worse. Moreover, there is limited information on levels of POPs in seafood and accordingly health risk assessments are hampered.

The aim of the present study was integrating a high consumption rate of shellfish and their contamination degree of a selected number of POPs, to determine residual concentrations of OCPs, PCBs, PBDEs, HBCDDs, and MeO-PBDEs in shellfish from a few locations along the eastern coastline of China. In addition, the potential sources and the extent of pollution are discussed.

## Materials and methods

### Samples

**Fig. 1:**
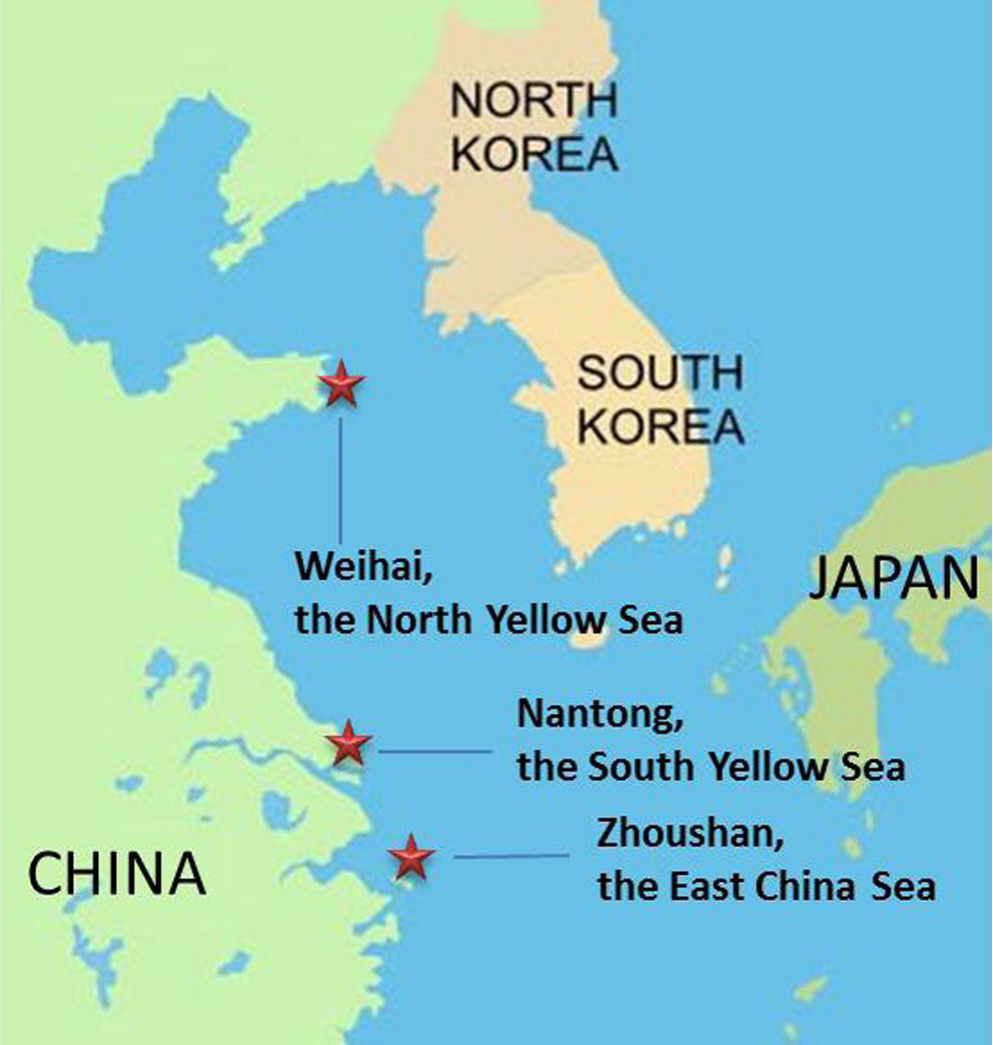
Sampling locations of shellfish for the present study

The shellfish were collected from three locations from eastern China. The sampling information is shown in Fig. 1 and Table S1. Blue mussels (*Mytilus edulis*) (ME) were taken from the North Yellow Sea, Weihai city (WH) and the East China Sea, Zhoushan city (ZS) in July 2010 and 2011, respectively. The blue mussels from ZS were collected from natural (ZS1) and cultivated source (ZS2). Three kinds of clams which are *Cyclina sinensis* (CS), *Ruditapes philippinarum* (RP), and *Sinonovacula constricta* (SC) were taken from the south Yellow Sea, Nantong city (NT) in July 2011. The clams, CS and RP, are the predominant clam species in the Yangtze River Estuary. All samples were packed in aluminum foil and kept frozen at −20 °C until the start of analysis.

### Chemicals

All solvents and chemicals used were of highest commercially available quality. Authentic reference standards of dichlorodiphenyltrichloroethanes (DDTs), including 4,4′-DDT, 4,4′-DDE, 4,4′-DDD, 2,4′-DDT, 2,4′-DDE, and 2,4′-DDD; HCHs, including α-HCH, β-HCH, γ-HCH, δ-HCH, and ε-HCH; hexachlorobenzene (HCB); mirex; α-endosulfan; and β-endosulfan were purchased as a mixture from Larodan Fine Chemicals (Malmö, Sweden). The PCB congeners: CB-28, CB-52, CB-101, CB-118, CB-138, CB-153, and CB-180, and PBDE congeners: BDE-47, BDE-85, BDE-90, BDE-99, BDE-100, BDE-153, BDE-154, and BDE-183, used as external standards were purchased from Larodan Fine Chemicals and LGC Promochem (Wesel, Germany), respectively. MeO-PBDEs (including 6-MeO-BDE-47, 2′-MeO-BDE-68, 6-MeO-BDE-85, 6-MeO-BDE-90, 6-MeO-BDE-99, 2-MeO-BDE-123, and 6-MeO-BDE-137) were synthesized in-house (Marsh et al. 2003; Marsh et al. 2005). Technical HBCDD was obtained from the Dead Sea Bromine Group (Israel). The full name of DDTs, PCBs, PBDEs, and MeO-PBDEs and their abbreviations are listed in Supplemental Material. Silica gel (0.063–0.2 mm) from Merck (Darmstadt, Germany) was activated at 300 °C in oven overnight before use.

### Extraction and cleanup

The shellfish meat was homogenized as an individual pool (*n* = 30) for each species and sampling site. The samples (10 g) were extracted as described elsewhere (Jensen et al. 2009), except that n-hexane was exchanged for cyclohexane due to its lower toxicity (for detailed description, see Supplemental Material). The lipid weight was determined gravimetrically for all the extracted samples after solvent removal. CB-200 (30 ng), CB-207 (1 ng), BDE-138 (1 ng), and 4-MeO-BDE-121 (1 ng) were added to the samples and blanks as surrogate standards. Lipids were removed by concentrated sulfuric acid (1 mL/0.1 g lipid) treatment, a procedure that was repeated once. After sulfuric acid treatment, the samples were further cleaned up on two different types of chromatographic columns. The first column was packed with activated silica (0.9 g) impregnated with concentrated sulfuric acid (2:1 *w*/*w*). The column was conditioned with cyclohexane (10 mL), and the analytes were eluted with dichloromethane (15 mL). The second column was packed with silica gel (0.5 g). It was pre-eluted with dichloromethane (5 mL) and then the analytes were eluted with dichloromethane (10 mL). The solvent volume was reduced by a gentle flow of nitrogen gas and changed to n-hexane. Prior to instrumental analysis, CB-189 (2 ng) and BDE-139 (1 ng) were added as volumetric standard. The final volumes were 0.5 mL for DDT analysis and 0.1 mL for the other compounds.

### Instrumental analysis

The analysis of PCBs and pesticide mixture was performed on a Varian 450 gas chromatogram equipped with electron capture detector (GC-ECD) maintained at 360 °C and a Varian CP-8400 autosampler. The injector (1 μL) was operated in the splitless mode, and the temperature for the injector was 260 °C. The non-polar Varian CP-Sil 8 CB (25 m × 150 μm × 0.12 μm) column (Middleburg, the Netherlands) was used. Helium was used as a carrier gas and nitrogen as the makeup gas. The column oven temperature was programmed from 80 °C for 1 min, 20 °C/min to 300 °C and held constant for 5 min.

The PBDEs, MeO-PBDEs, and HBCDDs were analyzed by gas chromatography-mass spectrometry (GC-MS) in selected ion monitoring mode (bromide ions m/z 79 and 81) and were identified by retention time using authentic reference standards. Automated 1 μL injections with a CTC GC Pal autosampler were conducted on a Varian 450-GC connected to a Varian 320-MS. A programmable temperature vaporizing (PTV) injector was used with a DB-5 HT capillary column (15 m × 250 μm × 0.1 μm) from J&W Scientific (Folsom, USA). Helium was used as carrier gas at a set constant flow of 1.2 mL/min. The ion source temperature was 200 °C and the transfer line temperature to 290 °C. Methane (scientific 5.5, AGA Stockholm, Sweden) was used as reagent gas. The oven was programmed as follows: 55 °C for 2 min, 15 °C/min to 320 °C and held constant for 5 min. The PTV injector temperature was 280 °C with a splitless mode. The PTV injector was also pressure programmed with a pressure pulse of 10 psi for 0.5 min upon injection.

### Quality control

One procedure blank was run for each batch of six samples to assess any potential contamination during laboratory work. Except for BDE congeners, no other analyte was present in blank samples. Three duplicates from each location and species were analyzed to determine the analytical precision. The range of recoveries (mean value ± standard deviation) for surrogate standards CB-207, BDE-138, and 4-MeO-BDE-121 were 79–115 % (103 ± 8 %), 106–118 % (112 ± 7 %), and 61–101 % (88 ± 19 %), respectively. All external calibration curves have a good correlation coefficient. Limit of detection was defined as three times the signal-to-noise (S/N) in the chromatogram and limit of quantification (LOQ) as ten times the S/N. If the blank samples were contaminated, the LOQ was defined as three times the average amount found in the procedure blanks. More information on LOQ is given in Supplementary Material and in Table S5.

## Results

**Fig. 2:**
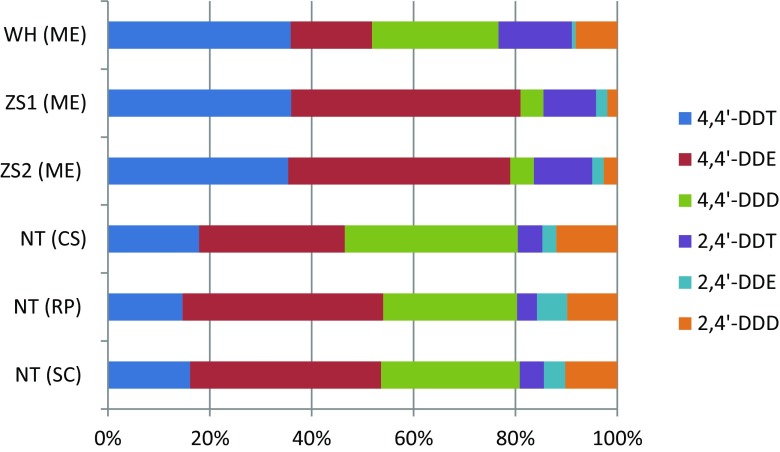
Patterns of DDTs in *Mytilus edulis* (*ME*), *Cyclina sinensis* (*CS*), *Ruditapes philippinarum* (*RP*), and *Sinonovacula constricta* (*SC*) from Weihai (*WH*), Zhoushan (*ZS*), and Nantong (*NT*). *ZS1* natural mussel and *ZS2* cultivated mussel

**Fig. 3:**
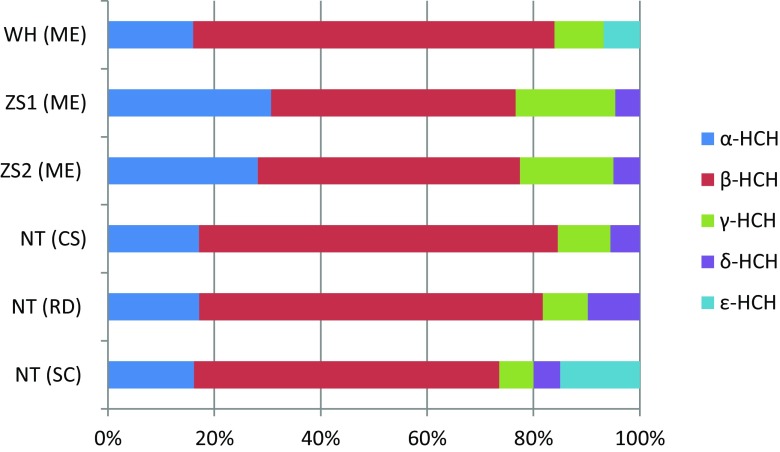
Patterns of HCHs in *Mytilus edulis* (*ME*), *Cyclina sinensis* (*CS*), *Ruditapes philippinarum* (*RP*), and *Sinonovacula constricta* (*SC*) from Weihai (*WH*), Zhoushan (*ZS*), and Nantong (*NT*). *ZS1* natural mussel and *ZS2* cultivated mussel

**Fig. 4:**
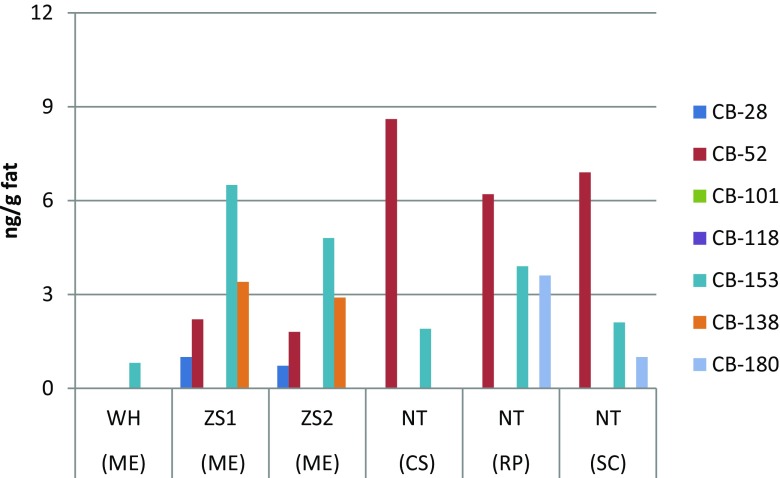
Concentrations of PCBs in *Mytilus edulis* (*ME*), *Cyclina sinensis* (*CS*), *Ruditapes philippinarum* (*RP*), and *Sinonovacula constricta* (*SC*) from Weihai (*WH*), Zhoushan (*ZS*), and Nantong (*NT*). *ZS1* natural mussel and *ZS2* cultivated mussel

**Fig. 5:**
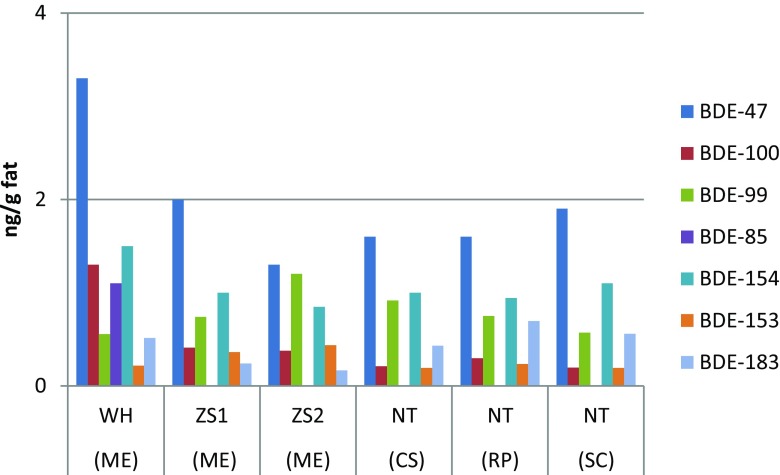
Concentrations of PBDEs in *Mytilus edulis* (*ME*), *Cyclina sinensis* (*CS*), *Ruditapes philippinarum* (*RP*), and *Sinonovacula constricta* (*SC*) from Weihai (*WH*), Zhoushan (*ZS*), and Nantong (*NT*). *ZS1* natural mussel and *ZS2* cultivated mussel

**Fig. 6:**
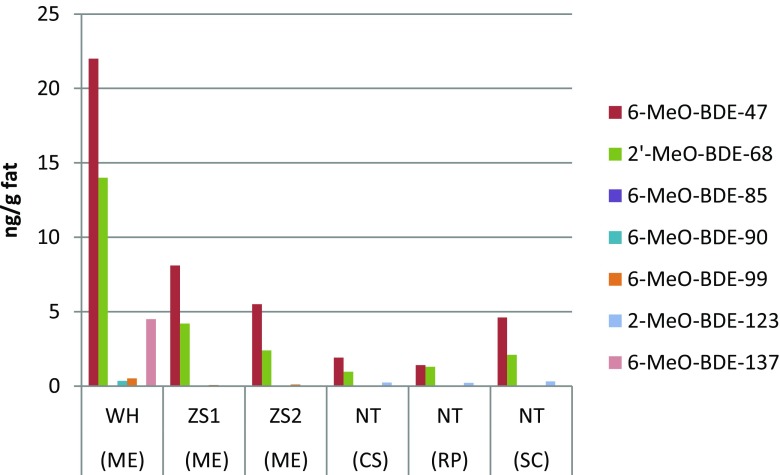
Concentrations of MeO-PBDEs in *Mytilus edulis* (*ME*), *Cyclina sinensis* (*CS*), *Ruditapes philippinarum* (*RP*), and *Sinonovacula constricta* (*SC*) from Weihai (*WH*), Zhoushan (*ZS*), and Nantong (*NT*). *ZS1* natural mussel and *ZS2* cultivated mussel

Mean concentrations of the individual main chlorinated and brominated organic analytes from the DDTs, HCHs, PCBs, PBDEs, HBCDD, and MeO-PBDEs in shellfish are presented in Table 1. Detailed concentration data of all the analytes are given in the Tables S2–4 and S6 of the Supplementary Material, including sum data of DDTs, HCHs, PCBs, PBDEs, and MeO-PBDEs. The relative contribution of the main three 4,4′-DDTs and the 2,4′-DDTs are given from each sampling site in Fig. 2. Similarly, the HCH isomer relative distribution in the sampled matrices is shown in Fig. 3, while the congener concentration patterns of PCBs, PBDEs, and MeO-PBDEs are presented in Figs. 4–6, respectively.

**Table 1:** Concentrations (ng/g fat) of some prioritized polychlorinated and brominated organic pollutants in *Mytilus edulis* (ME), *Cyclina sinensis* (CS), *Ruditapes philippinarum* (RP), and *Sinonovacula constricta* (SC) from Weihai (WH), Zhoushan (ZS), and Nantong (NT); ZS1 and ZS2 are natural and cultivated mussel, respectively

Compound	WH (ME) (*n* = 3)		ZS1 (ME) (*n* = 3)		ZS2 (ME) (*n* = 3)		NT (CS) (*n* = 3)		NT (RP) (*n* = 3)		NT (SC) (*n* = 3)	
	Mean	S.D.	Mean	S.D.	Mean	S.D.	Mean	S.D.	Mean	S.D.	Mean	S.D.
Lipid (%)	2.6	0.11	1.6	0.19	2.0	0.27	1.6	0.050	0.97	0.030	2.2	0.090
4,4′-DDT	1100	99	160	8.0	130	15	100	2.4	67	0.74	190	4.3
4,4′-DDE	490	25	200	9.8	160	13	160	6.1	180	3.7	440	5.3
4,4′-DDD	760	30	20	1.7	17	1.3	190	6.8	120	1.9	320	3.6
α-HCH	0.66	0.028	1.8	0.22	1.6	0.14	0.99	0.15	1.3	0.57	0.65	0.070
β-HCH	2.8	0.21	2.7	0.29	2.8	0.19	3.9	0.50	4.9	0.19	2.3	0.23
γ-HCH	0.38	0.017	1.1	0.053	1.0	0.022	0.57	0.073	0.64	0.18	0.26	0.062
β-endosulfan	1.8	0.22	0.78	0.14	0.70	0.056	1.0	0.15	1.9	0.16	0.35	0.058
HCB	1.1	0.45	2.4	0.085	1.7	0.12	1.5	0.48	3.4	0.045	3.6	0.31
CB-153	0.81	0.096	6.5	0.77	4.8	0.74	1.9	0.24	3.9	0.24	2.1	0.43
BDE-47	3.3	0.54	2.0	0.24	1.3	0.24	1.6	0.50	1.6	0.22	1.9	0.46
HBCDD	40	7.8	32	7.1	21	8.3	42	8.1	34	4.5	38	0.34
6-MeO-BDE-47	22	2.1	8.2	2.4	5.5	1.8	1.9	0.65	1.3	0.35	4.6	0.41
2′-MeO-BDE-68	14	1.6	4.2	1.3	2.4	1.0	0.96	0.32	1.3	0.34	2.1	0.17

## Discussion

It is notable that the three 4,4′-DDTs are present at high level in shellfish from all sites in the present study, making up approximately 80 % of the total concentrations of DDTs. The data confirm recent or even ongoing releases of 4,4′-DDT (cf. below). However, another interesting result is the relatively high levels of HBCDD independent of sampling site and species analyzed (Table 1). The concentrations of HBCDD are coming up as the second most important pollutant in any of the shellfish species, being higher than even the sum concentrations of any of the other pollutants analyzed, except the DDTs. This observation is supported by the levels reported of HBCDD in oysters and blue mussels sampled from coastal areas of Japan (Table 2) (Ueno et al. 2010). Shellfish contaminant levels, as determined in the present study are put in perspective to reports from other sites in Table 2. Unfortunately, it is not possible to compare all reported data due to different basis for presenting the levels in the international literature, but for those studies permitting recalculations to present either wet weight or lipid weight based concentrations, data are presented in Table 2. The results of the present study are put in perspective for each of the contaminant congener group of pollutants or of individual contaminants, which are discussed groupwise, below.

**Table 2:** Concentration ranges of chlorinated and brominated pollutants in different shellfish species around the world

Location	Sampling year	Species	Unit (ng/g)	Lipid content (%)	Compounds											Reference
					4,4′-DDT	4,4′-DDE	4,4′-DDD	2,4′-DDT	DDTs^a^	HCHs^b^	HCB	CB-153	7CBs^c^	BDE-47	HBCDD	
Weihai, China	2010	Blue mussel	w.w.	2.6	29	13	20	11		0.11	0.029	0.021	0.021	0.086	1.0	Present study
			l.w.		1,100	490	760	440		4.1	1.1	0.81	0.81	3.3	40	
Zhoushan, China	2011	Blue mussel	w.w.	1.6–2.0	2.6	3.2	0.32–0.34	0.67–0.80		0.09–0.11	0.03–0.04	0.10	0.21	0.03	0.42–0.51	Present study
			l.w.		130–160	160–200	17–20	42–46		5.7–5.8	1.5–1.7	4.8–6.5	11–15	1.3–2.0	21–32	
Nantong, China	2011	Clam	w.w.	0.97–2.2	0.65–4.2	1.8–9.7	1.2–7.0	0.17–1.2		0.07–0.09	0.03–0.08	0.03–0.05	0.13–0.22	0.02–0.04	0.33–0.84	Present study
			l.w.		67–190	160–440	120–320	18–56		4.0–7.6	2.4–3.4	1.9–3.9	10–14	1.6–1.9	34–42	
Shengsi, China	2004	Green mussel	l.w.	0.88					1,500	24	5.0			3.1		(Ramu et al. 2007b)
Chongming, China	2004	Blue mussel	l.w.	0.79					13,000	96	4.2			6.1		(Ramu et al. 2007b)
Jiaozhou Wan, China	2004	Blue mussel	l.w.	0.78					10,000	79	5.5			5.9		(Ramu et al. 2007b)
Qiantang River, China	2006	Mussel	w.w.	3.0	6.8	15	3.8	1.20		12.2						(Zhou et al. 2008)
Yangtze Estuary, China	2002	Clam	w.w.	1.6–1.9	2.6–12	3–8.9	n.d.–9.8^d^	1–15		2–4.6						(Yang et al. 2006)
Pearl River Delta, China	2001–2002	Green mussel	l.w.		n.d.–41^d^	n.d.–104^d^	n.d.–200^d^			n.d.–1.04^d^						(Fang 2004)
Mariculture zones, Hong Kong	2002	Green mussel	l.w.		0.90–210	5.8–1,400	0.7–230			12–780	26–430	0.90–118	44–460			(So et al. 2005)
Coastal area of Hong Kong	2004	Green mussel	l.w.	0.64–1.7					720–58,000	3.8–18	<1					(Ramu et al. 2007b)
East coast, India	2004	Green mussel	l.w.	0.37–2.4					380–640	7.2–230	<1					(Ramu et al. 2007b)
Coastal area of Korea	2005	Blue mussel	l.w.	0.72–2.3					24–400	1.1–82	0.31–50			0.020–3.3		(Ramu et al. 2007a)
Coastal areas of Japan		Oysters and blue mussel	l.w.		6.9–760	18–570	5.3–350			n.d.–44^d^		3.8–420	16–1,900	1.6–23	12–5,200	(Ueno et al. 2010)
Ionian Sea, Italy	2008	Mussel	w.w.		n.d.^d^	n.d.–5.4^d^	n.d.–0.34^d^	n.d.^d^				n.d.–31^d^	n.d.–78^d^			(Giandomenico et al. 2013)
Scheldt estuary, Netherlands and Belgium	2010	Blue mussel	w.w.	2.0–3.7	0.09–1.2	10–39	3.5–16			0.83–1.8	0.09–0.76	51–360	120–760	0.28–5.2		(Van Ael et al. 2012)
Adriatic coast, Croatia	2003–2008	Blue mussel	w.w.						0.56–2.0	0.15–0.62	0.02–0.17		0.28–2.1			(Kozul et al. 2011)
Baltic Sea, Finland	2007	Clam	w.w.		0.13–0.47	1.0–3.0	0.23–1.0			0.24–0.49	0.07–0.15	0.56–1.6	1.7–4.3			(Pikkarainen 2007)
Usuk, Greenland	2000	Blue mussel	l.w.	2.0	6.1	11	5.1			13	4.1	7.1	15	5.1		(Christensen et al. 2002; Glasius et al. 2005)

^a^Sum of 4,4′-DDT, 4,4′-DDE, and 4,4′-DDD

^b^Sum of α-HCH, β-HCH, and γ-HCH

^c^Sum of CB-28, CB-52, CB-101, CB-118, CB-138, CB-153, and CB-180

^d^n. d. not detected

### Organohalogen pesticides in shellfish

#### DDTs

The patterns of the DDTs in the shellfish, in the present study, are shown in Fig. 2 and Table S2. The mean concentrations of 4,4′-DDT in shellfish from the three sites were quite similar to other studies in the waters close to China, Japan, India, and Republic of Korea (Ramu et al. 2007b; Ramu et al. 2007a; Yang et al. 2006; Zhou et al. 2008), except for levels reported in green mussels from Hong Kong (Ramu et al. 2007b), showing 10 to a 100 times higher ∑DDT concentrations (Table 2). Contrast to the high 4,4′-DDE concentration in mussel from Zhoushan, 4,4′-DDD levels were comparable to 4,4′-DDE’s level in clam from Nantong. It might be due to the species-specific lifestyle. As mentioned, mussels are stick in the rock but clams live in the sediment where 4,4′-DDT is prior to degrade to form 4,4′-DDD under anaerobic condition. The levels of the three 4,4′-DDTs, as reported in shellfish from other sites, primarily Europe, were similar or lower than those from eastern Asia (Giandomenico et al. 2013; Pikkarainen 2007; Van Ael et al. 2012), c.f. Table 2.

The occurrence of 2,4′-DDT was in the expected range (0.21–0.24) for technical DDT products but with one sampling site, Weihai, showing a higher ratio (0.29) that is difficult to explain unless there were current discharges of another, less well known, DDT product. The ratio of 4,4′-DDT/(4,4′-DDT + 4,4′-DDE + 4,4′-DDD) indicated that there were fresh or at least recent input of DDT in Weihai and the Zhoushan areas (ratios are 0.42–0.47), while less so in the Nantong area (ratios here are 0.21–0.23).

The results on the DDTs from the Weihai and Zhoushan areas indicate that DDT is still used in China. DDT was banned as a pesticide in 1983 in China but it has been continuously manufactured for some non-agricultural purposes, i.e., malaria control, export, painting of boat hulls (ban implemented 2009), and synthesis of dicofol (UNEP 2007; Wong et al. 2005). It is reported that the majority of DDT produced has been used for dicofol synthesis since 1988 (Qiu et al. 2005). The ratio of 2,4′-DDT/4,4′-DDT was applied to explore the source of DDT (Qiu et al. 2005). The low 2,4′-DDT/4,4′-DDT ratio in this study implies the newly input DDTs could not only be attribute to the dicofol usage.

#### HCHs

The relative concentrations of five HCH isomers, determined for all shellfish analyzed, showed a rather even distribution between sampling sites, as visualized in Fig. 3 and with concentration data shown in Table S2. The high abundance of β-HCH indicates previous use of technical HCH, i.e., a mixture of the isomers α-HCH (60–70 %), β-HCH (5–12 %), γ-HCH (10–12 %), δ-HCH (6–10 %), and ɛ-HCH (3–4 %). However, β-HCH was the most persistent, due to its chemical stability, of the HCH isomers and accordingly the isomer to be expected in the highest concentrations if technical HCH was the source. The ratio of α-HCH/γ-HCH detected in the present study ranged from 1.0 to 2.5, which was lower than technical HCH which varies between 4 and 7 (Walker et al. 1999). This result indicated other sources, i.e., lindane (99 % γ-HCH) has been continuously used as an insecticide after technical HCH was banned. Comparing ∑(α-, β-, γ-HCH) levels in shellfish from around the world, it is notable that the present concentrations were in the lower end in relation to data from east Asian waters (Ramu et al. 2007b) and also compared to blue mussels from Greenland (Glasius et al. 2005) (Table 2). However, comparing ∑(α-, β-, γ-HCH) levels presented herein with the European sampling sites (Kozul et al. 2011; Van Ael et al. 2012) indicated similar or only slightly higher levels (Table 2). It is reasonable to conclude that the high proportion of β-HCH together with low ratio of α-HCH/γ-HCH indicates that HCHs profile in shellfish was due to contamination of technical HCH together with lindane.

#### Endosulfan

The α- and β-endosulfan were determined in all samples except for α-endosulfan in Weihai mussels (Table S2). The concentration of endosulfan present in this study varied from 0.57 to 2.3 ng/g fat, which is lower than another study on mollusks in China (Liu et al. 2010). β-Endosulfan dominated in the shellfish sampled independent of sampling site, accounting for 100, 71, and 50 % for Weihai, Zhoushan, and Nantong area, respectively. Technical endosulfan consists of 70 % α-endosulfan and 30 % β-endosulfan. It is worth to note that the β-endosulfan concentrations were similar or higher than those of the HCH isomers and HCB, assessed and reported herein (Table 1 and S2).

#### Mirex

Mirex was only detected in shellfish from Nantong in a concentration range of 0.6–2.5 ng/g fat (Table S2). This is similar as the one determined for β-endosulfan (cf. above). The occurrence of mirex in the shellfish from the Nantong area is not surprising since most, if not all, of the mirex manufacturing is located to the Jiangsu Province (Wang et al. 2010a). Accordingly, mirex reported herein might be from its production or application as a pesticide. China is the country that has suffered from termite damage most severely in the world, especially in the south. However, on a regional scale, the level of mirex determined in the present study was low compared with another study (Jia et al. 2011). Jia et al. measured mirex in oyster in north of China with a mean concentration of 2 ng/g w.w. China has started to substitute mirex for dechlorane plus (Shen et al. 2010).

### Industrial organohalogens in shellfish

#### HCB

HCB levels in shellfish as reported in the present study (1.1–3.6 ng/g fat) are similar to most shellfish levels previously reported as summarized in Table 2 except for a couple of studies (Ramu et al. 2007b; So et al. 2005). Particularly, the latter was reporting on high HCB concentrations in green mussels from Hong Kong (26–430 ng/g fat) (So et al. 2005).

The HCB may come from several sources, i.e., if used as a pesticide, general combustion (Wang et al. 2010b), industrial by-product, and its use as a starting material for production of pentachlorophenol (PCP) and pentachlorophenol-Na (PCP-Na) (Wong et al. 2005). Qiu and coworkers has reported positive relation between concentration of pentachloroanisole, a metabolite of PCP, and HCB in fish samples from Taihu lake (Qiu et al. 2012). China has stopped producing and using HCB since 2004 (UNEP 2007). Nevertheless, temporal trend of HCB and PCP is needed for monitoring the development of these OCPs in the Chinese environment.

#### PCBs

PCB concentrations are presented in the diagram, Fig. 4, indicating overall low concentrations in the shellfish. It seems that there was a somewhat higher concentration of CB-52 in the Nantong area shellfish, while CB-153 was more evenly distributed between the sites and at a slightly lower concentration (Fig. 4). Tabulated concentrations are presented in Table S4. The PCB concentrations were in the lower end of all global reports used for reference purposes herein (Table 2). The data confirm limited uses of PCBs in China.

#### HBCDDs and PBDEs

As shown in Table 1, relatively high and evenly distributed HBCDD concentrations (21 to 42 ng/g fat) were determined in the shellfish analyzed in the present study. Still, these levels were lower than those reported for oysters and blue mussels from coastal areas of Japan (Table 2) (Ueno et al. 2010). In contrast, the presently analyzed shellfish had 2–4 times higher HBCDD levels than blue mussel from Sweden (NRM 2012) and reported in fish from Taihu Lake (Qiu et al. 2012). Due to the analytical methodology (GC-MS), it is unfortunately not possible to report the HBCDD isomer pattern. The data points at regional contamination of HBCDD but no details are yet known to us.

The PBDEs concentrations determined in shellfish in the present study were rather low (Fig. 5, Table S4), with BDE-47 showing the highest levels among the PBDEs. The levels reported in this study were similar to those reported in another study in China (Table 2) (Ramu et al. 2007b). Other congeners with concentrations in descending order were BDE-154, BDE-99, BDE-153, BDE-100, and BDE-183. The pattern observed was consistent with another study (Gao et al. 2009).

The reason for composition of PBDE congeners found in this study is not known. It could be explained by commercial PentaBDE product. However, it could also have been influenced by transformation and debromination. For instance, BDE-99 can be transformed to BDE-47 while BDE-154 is transformed from BDE-183 in common carp (Stapleton et al. 2004). Other studies show that BDE-47 could be formed through microbial reductive debromination (He et al. 2006) or photochemical degradation (Fang et al. 2008). So far, there is a lack of knowledge on PBDE metabolism and transformation in shellfish.

### Natural compounds in shellfish

#### MeO-PBDEs

The two naturally produced compounds, 6-MeO-BDE-47 and 2′-MeO-BDE-68, were detected in 100 % of the shellfish samples, independent of sampling location (Fig. 6, Table S6). The highest concentration of MeO-PBDEs in shellfish was observed in blue mussels from Weihai and Zhoushan, while lower in the other shellfish species from Nantong. It is not possible to state if this is a species difference or a difference in the environmental concentrations at the different sites. The concentrations of MeO-PBDEs in this study were consistent with the other in bivalve from Liaohe Bay in China (16 ng/g fat) (Zhang et al. 2010). The level of MeO-PBDEs was comparable to those in mussel from Canada (14 ng/g d.w.) (Kelly et al. 2008) and was lower than those in the Baltic Sea (160–420 ng/g fat) (Lofstrand et al. 2011).

Besides 6-MeO-BDE-47 and 2′-MeO-BDE-68, also several other *ortho*-MeO-substituted MeO-PBDEs were detected and quantified (Table S6). MeO-PBDEs reported in marine biota species are mostly *ortho*-substituted and are considered as natural product originating from alga or its associated microflora (Malmvarn et al. 2005). Qiu et al. ((2012) reported that 6-MeO-BDE-47 in fish from Taihu Lake may come from cyanobacteria. The MeO-PBDE level measured in this study was similar to those reported in fish (Qiu et al. 2012).

## Concluding remarks

The results from this study clearly show a severe pollution situation regarding the DDTs in the shellfish from the Yellow Sea and the East China Sea. The relatively high HBCDD concentrations in shellfish from all three sampling locations must be followed up by screening in other wildlife species and search for sources of contamination. HBCDD is indeed proposed as a new POP according to the Stockholm Convention, indicating the importance of controlling also this BFR. Further, it may be of interest to look closer into also the POPs not measured herein.

It is a serious obstacle that the scientific literature is not better coordinated in relation to reporting contaminant levels (Table 2). In this table, it is necessary to compare data both on wet and lipid weight basis due to the lack of proper reporting of lipid weight. Data from several studies had to be recalculated to enable comparison to wet weight concentrations since the original data are reported on dry weight. As authors of this article, we ask for actions in this context, possibly via scientist community development of weight of evidence for exposures to pollutants in wildlife.

The data presented herein include several shellfish species but it seems reasonable to propose more work on blue mussels since this is indeed a common species for environmental contamination levels around the world. The use of blue mussels will allow comparisons between truly wild mussels and cultivated blue mussels. This may be a possibility for locating point sources of POPs.

## Electronic supplementary material

Below is the link to the electronic supplementary material.

ESM1 (DOC 177 kb)
