# Supplementary material for: Chlorinated and brominated organic pollutants in shellfish from the Yellow Sea and East China Sea
Source: Environ Sci Pollut Res Int. 2014 Jun 25;22(3):1713–22. doi: 10.1007/s11356-014-3198-8 (PMC6684575; doi:10.1007/s11356-014-3198-8)
Supplement: Supplementary file 1 — (DOC 177 kb) [file 11356_2014_3198_MOESM1_ESM.doc]

**Chlorinated and brominated organic pollutants in shellfish from the Yellow Sea and East China Sea**

**Ge Yin^1^ Lillemor Asplund^1^ Yanling Qiu^2^ Yihui Zhou^2^ Hua Wang^2^ Zongli Yao^3^ Jianbin Jiang^4^ and Åke Bergman^1,2^**

1. Department of Materials and Environmental Chemistry, Stockholm University, 10691 Stockholm, Sweden; E-mail: ge.yin@mmk.su.se

2. College of Environmental Science and Engineering, Tongji University, Shanghai 200092, China

3. East China Sea Fisheries Research Institute, Chinese Academy of Fishery Sciences, Shanghai 200090, China

4. Tongzhou Fisheries Technical Instruction Station of Nantong, Nantong 226300, China

**Supplementary Material Content**

Chemicals and abbreviations

Extration method

Table S1. Detailed sample and sampling information.

Table S2. Concentrations (ng/g fat) of all the organochlorine pesticides (OCPs) analysed for in *Mytilus edulis* (ME), *Cyclina sinensis* (CS)*, Ruditapes philippinarum* (RP) *and Sinonovacula constricta* (SC) from Weihai (WH), Zhoushan (ZS) and Nantong (NT), ZS1 and ZS2 were natural and cultivated mussel, respectively.

Table S3. Concentrations (ng/g fat) of individual PCB congeners in *Mytilus edulis* (ME), *Cyclina sinensis* (CS)*, Ruditapes philippinarum* (RP) *and Sinonovacula constricta* (SC) from Weihai (WH), Zhoushan (ZS) and Nantong (NT), ZS1 and ZS2 were natural and cultivated mussel, respectively.

Table S4. Concentrations (ng/g fat) of individual PBDE congeners and HBCDDs in *Mytilus edulis* (ME), *Cyclina sinensis* (CS)*, Ruditapes philippinarum* (RP) *and Sinonovacula constricta* (SC) from Weihai (WH), Zhoushan (ZS) and Nantong (NT), ZS1 and ZS2 were natural and cultivated mussel, respectively.

Table S5. Limit of quantification (ng/g fat) for PBDEs in *Mytilus edulis* (ME), *Cyclina sinensis* (CS)*, Ruditapes philippinarum* (RP) *and Sinonovacula constricta* (SC) from Weihai (WH), Zhoushan (ZS) and Nantong (NT), ZS1 and ZS2 were natural and cultivated mussel, respectively.

Table S6. Concentrations (ng/g fat) of individual MeO-PBDE congeners in *Mytilus edulis* (ME), *Cyclina sinensis* (CS)*, Ruditapes philippinarum* (RP) *and Sinonovacula constricta* (SC) from Weihai (WH), Zhoushan (ZS) and Nantong (NT), ZS1 and ZS2 were natural and cultivated mussel, respectively.

**Chemicals and abbreviations**

The full names (abbreviations) of the DDTs discussed in the article are: 2,2-bis(4-chlorophenyl)-1,1,1-trichloroethane (4,4’-DDT); 2,2-bis(4-chlorophenyl)-1,1-dichloroethene (4,4’-DDE); 2,2-bis(4-chlorophenyl)-1,1-dichloroethane (4,4’-DDD); 2-(2-chlorophenyl)-2-(4-chlorophenyl)-1,1,1-trichloroethane (2,4’-DDT); 2-(2-chlorophenyl)-2-(4-chlorophenyl)-1,1-dichloroethene (2,4’-DDE) and 2-(2-chlorophenyl)-2-(4-chlorophenyl)-1,1-dichloroethane (2,4’-DDD).

The polychlorinated biphenyl (PCB) congeners discussed are: 2,4,4’-trichlorobiphenyl (CB-28); 2,2’,5,5’-tetrachlorobiphenyl (CB-52); 2,2’,4,5,5’-pentachlorobiphenyl (CB-101); 2,3’,4,4’,5-pentachlorobiphenyl (CB-118); 2,2’,3,4,4’,5’-hexachlorobiphenyl (CB-138); 2,2’,4,4’,5,5’-hexachlorobiphenyl (CB-153); 2,2’,3,4,4’5,5’-heptachlorobiphenyl (CB-180); 2,3,3’,4,4’,5,5’-heptachlorobiphenyl (CB-189, volumetric standard); 2,2’,3,3’4,5,6,6’-octachlorobiphenyl (CB-200, surrogate standard) and 2,2’,3,3’,4,4’,5,6,6’-nonachlorobiphenyl (CB-207, surrogate standard).

The polybrominated diphenyl ether (PBDE) congeners are 2,2’,4-tribrominated diphenyl ether (BDE-28); 2,2’,4,4’-tetrabrominated diphenyl ether (BDE-47); 2,2’,3,4,4’-pentabrominated diphenyl ether (BDE-85); 2,2’,4,4’,5-pentabrominated diphenyl ether (BDE-99); 2,2’,4,4’,6-pentabrominated diphenyl ether (BDE-100); 2,2’,3,4,4’,5’-hexabrominated diphenyl ether (BDE-138, surrogate standard); 2,2’,3,4,4’,6-hexabrominated diphenyl ether (BDE-139, volumetric standard); 2,2’,4,4’,5,5’-hexabrominated diphenyl ether (BDE-153); 2,2’,4,4’,5,6’-hexabrominated diphenyl ether (BDE-154) and 2,2’,3,4,4’,5’,6-heptabrominated diphenyl ether (BDE-183).

The methoxylated PBDE (MeO-PBDE) discussed are: 6-methoxy-2,2’,4,4’-tetrabrominated diphenyl ether (6-MeO-BDE-47); 2’-methoxy-2,3’,4,5’-tetrabrominated diphenyl ether (2’-MeO-BDE-68); 6-methoxy-2,2’,3,4,4’-pentabrominated diphenyl ether (6-MeO-BDE-85); 6-methoxy-2,2’,3,4’,5-pentabrominated diphenyl ether (6-MeO-BDE-90); 6-methoxy-2,2’,4,4’,5-pentabrominated diphenyl ether (6-MeO-BDE-99); 4’-methoxy-2,3’,4,5’,6-pentabrominated diphenyl ether (4’-MeO-BDE-121, Surrogate Standard); 2-methoxy-2’,3,4,4’,5-pentabrominated diphenyl ether (2-MeO-BDE-123) and 6-methoxy-2,2’,3,4,4’,5-hexabrominated diphenyl ether (6-MeO-BDE-137).

**Extraction method**

The samples were weighted to get the fresh weight (about 10 g, see table S1) and then transferred to a centrifuge tube containing isopropanol (IPR, 25 ml). The analytes were extracted with cyclohexane/diethyl ether (*c-*Hxn/DEE, 3:1, v/v, 20 ml). After homogenization and centrifugation, the extracts were poured into a 150 ml separatory funnel. Re-extraction was done using IPR (10 ml) and *c-*Hxn/DEE (3:1, v/v, 40 ml) and the extracts were combined in the separatory funnel. The extracts were then washed with a mixture of sodium chloride (0.9%) in hydrochloric acid (0.2 mol/L HCl, 50 ml), inverted 30 times and waited for phase separation. After 1 h, the water phase was removed to an E-flask. The funnel was washed with the HCl solution (20 ml) again and separation procedure repeated. Finally, the organic phase was decanted into a pre-weighted beaker. The beaker was left in the fume hood at room temperature overnight for evaporation until a stable lipid weight was reached.

Table S1. Detailed pooled sample (n=30) and sampling information for *Mytilus edulis* (ME), *Cyclina sinensis* (CS)*, Ruditapes philippinarum* (RP) *and Sinonovacula constricta* (SC) from Weihai (WH), Zhoushan (ZS) and Nantong (NT).

| Sampling site |  | Sampling date | Fresh weight (g) | | Lipid weight (g) | | Lipid (%) | Mean (%) | S.D. |
| --- | --- | --- | --- | --- | --- | --- | --- | --- | --- |
| YS (ME) 1 | | July 2010 | 11.252 |  | 0.3035 |  | 2.7 |  |  |
| YS (ME) 2 | | July 2010 | 11.376 |  | 0.2867 |  | 2.5 | 2.6 | 0.11 |
| YS (ME) 3 | | July 2010 | 11.370 |  | 0.2823 |  | 2.5 |  |  |
|  |  |  |  |  |  |  |  |  |  |
| ZS1 (ME) 1 | | July 2011 | 10.344 |  | 0.1744 |  | 1.7 |  |  |
| ZS1 (ME) 2 | | July 2011 | 10.633 |  | 0.1509 |  | 1.4 | 1.6 | 0.19 |
| ZS1 (ME) 3 | | July 2011 | 10.754 |  | 0.1922 |  | 1.8 |  |  |
|  |  |  |  |  |  |  |  |  |  |
| ZS1 (ME) 1 | | July 2011 | 10.424 |  | 0.1820 |  | 1.7 |  |  |
| ZS1 (ME) 2 | | July 2011 | 9.899 |  | 0.2011 |  | 2.0 | 2.0 | 0.27 |
| ZS1 (ME) 3 | | July 2011 | 10.620 |  | 0.2421 |  | 2.3 |  |  |
|  |  |  |  |  |  |  |  |  |  |
| NT (CS) 1 | | July 2011 | 9.924 |  | 0.1618 |  | 1.6 |  |  |
| NT (CS) 2 | | July 2011 | 10.605 |  | 0.1628 |  | 1.5 | 1.6 | 0.05 |
| NT (CS) 3 | | July 2011 | 10.349 |  | 0.1653 |  | 1.6 |  |  |
|  |  |  |  |  |  |  |  |  |  |
| NT (RP) 1 | | July 2011 | 9.829 |  | 0.0994 |  | 1.0 |  |  |
| NT (RP) 2 | | July 2011 | 11.160 |  | 0.1063 |  | 0.95 | 0.97 | 0.03 |
| NT (RP) 3 | | July 2011 | 10.209 |  | 0.0976 |  | 0.96 |  |  |
|  |  |  |  |  |  |  |  |  |  |
| NT (SC) 1 | | July 2011 | 10.594 |  | 0.2374 |  | 2.2 |  |  |
| NT (SC) 2 | | July 2011 | 10.681 |  | 0.2284 |  | 2.1 | 2.2 | 0.09 |
| NT (SC) 3 | | July 2011 | 10.484 |  | 0.2165 |  | 2.1 |  |  |

Table S2. Concentrations (ng/g fat) of all the organochlorine pesticides (OCPs) analysed for in *Mytilus edulis* (ME), *Cyclina sinensis* (CS)*, Ruditapes philippinarum* (RP) *and Sinonovacula constricta* (SC) from Weihai (WH), Zhoushan (ZS) and Nantong (NT), ZS1 and ZS2 were natural and cultivated mussel, respectively.

| Compound | WH (ME)  (n=3) | | ZS1 (ME)  (n=3) | | ZS2 (ME)  (n=3) | | NT (CS)  (n=3) | | NT (RP)  (n=3) | | NT (SC)  (n=3) | |
| --- | --- | --- | --- | --- | --- | --- | --- | --- | --- | --- | --- | --- |
|  | mean | S.D. | mean | S.D. | mean | S.D. | mean | S.D. | mean | S.D. | mean | S.D. |
| *Lipid (%)* | 2.6 | 0.11 | 1.6 | 0.19 | 2.0 | 0.27 | 1.6 | 0.050 | 0.97 | 0.030 | 2.2 | 0.090 |
| 4,4’-DDT | 1100 | 99 | 160 | 8.0 | 130 | 15 | 100 | 2.4 | 67 | 0.74 | 190 | 4.3 |
| 4,4’-DDE | 490 | 25 | 200 | 9.8 | 160 | 13 | 160 | 6.1 | 180 | 3.7 | 440 | 5.3 |
| 4,4’-DDD | 760 | 30 | 20 | 1.7 | 17 | 1.3 | 190 | 6.8 | 120 | 1.9 | 320 | 3.6 |
| 2,4’-DDT | 440 | 12 | 46 | 2.5 | 42 | 3.1 | 27 | 0.45 | 18 | 0.77 | 56 | 1.3 |
| 2,4’-DDE | 24 | 0.41 | 9.8 | 0.54 | 8.4 | 0.56 | 15 | 0.54 | 27 | 0.81 | 49 | 0.72 |
| 2,4’-DDD | 250 | 8.0 | 8.6 | 0.89 | 9.7 | 1.1 | 67 | 5.3 | 45 | 0.97 | 120 | 8.0 |
| ∑DDTs | 3100 |  | 440 |  | 370 |  | 560 |  | 460 |  | 1200 |  |
| α-HCH | 0.66 | 0.028 | 1.8 | 0.22 | 1.6 | 0.14 | 0.99 | 0.15 | 1.3 | 0.57 | 0.65 | 0.070 |
| β-HCH | 2.8 | 0.21 | 2.7 | 0.29 | 2.8 | 0.19 | 3.9 | 0.50 | 4.9 | 0.19 | 2.3 | 0.23 |
| γ-HCH | 0.38 | 0.017 | 1.1 | 0.053 | 1.0 | 0.022 | 0.57 | 0.073 | 0.64 | 0.18 | 0.26 | 0.062 |
| δ-HCH | n.d. |  | 0.27 | 0.0053 | 0.28 | 0.0079 | 0.22 | 0.048 | 0.74 | 0.070 | 0.20 | 0.0049 |
| ε-HCH | 0.28 | 0.0061 | n.d. |  | n.d. |  | n.d. |  | n.d. |  | 0.60 | 0.033 |
| ∑HCHs | 4.1 |  | 5.9 |  | 5.7 |  | 5.8 |  | 7.6 |  | 4.0 |  |
| HCB | 1.1 | 0.45 | 2.4 | 0.085 | 1.7 | 0.12 | 1.5 | 0.48 | 3.4 | 0.045 | 3.6 | 0.31 |
| α-endosulfan | n.d. |  | 0.59 | 0.17 | 0.94 | 0.18 | 0.42 | 0.18 | 0.41 | 0.081 | 0.22 | 0.0077 |
| β-endosulfan | 1.8 | 0.22 | 0.78 | 0.14 | 0.70 | 0.056 | 1.0 | 0.15 | 1.9 | 0.16 | 0.35 | 0.058 |
| ∑endosulfan | 1.8 |  | 1.4 |  | 1.6 |  | 1.4 |  | 2.3 |  | 0.57 |  |
| Mirex | n.d. |  | n.d. |  | n.d. |  | 1.6 | 0.21 | 2.5 | 0.32 | 0.62 | 0.064 |

Table S3. Concentrations (ng/g fat) of individual PCB congeners in *Mytilus edulis* (ME), *Cyclina sinensis* (CS)*, Ruditapes philippinarum* (RP) *and Sinonovacula constricta* (SC) from Weihai (WH), Zhoushan (ZS) and Nantong (NT), ZS1 and ZS2 were natural and cultivated mussel, respectively.

| Compound | WH (ME)  (n=3) | | ZS1 (ME)  (n=3) | | ZS2 (ME)  (n=3) | | NT (CS)  (n=3) | | NT (RP)  (n=3) | | NT (SC)  (n=3) | |
| --- | --- | --- | --- | --- | --- | --- | --- | --- | --- | --- | --- | --- |
|  | mean | S.D. | mean | S.D. | mean | S.D. | mean | S.D. | mean | S.D. | mean | S.D. |
| *Lipid (%)* | 2.6 | 0.11 | 1.6 | 0.19 | 2.0 | 0.27 | 1.6 | 0.050 | 0.97 | 0.030 | 2.2 | 0.090 |
| CB-28 | n.d. |  | 0.98 | 0.12 | 0.72 | 0.53 | n.d. |  | n.d. |  | n.d. |  |
| CB-52 | n.d. |  | 2.2 | 0.27 | 1.8 | 0.076 | 8.6 | 1.2 | 6.2 | 1.1 | 6.9 | 0.50 |
| CB-101 | n.d. |  | n.d. |  | n.d. |  | n.d. |  | n.d. |  | n.d. |  |
| CB-118 | n.d. |  | n.d. |  | n.d. |  | n.d. |  | n.d. |  | n.d. |  |
| CB-138 | n.d. |  | 3.4 | 0.22 | 2.9 | 0.09 | n.d. |  | n.d. |  | n.d. |  |
| CB-153 | 0.81 | 0.096 | 6.5 | 0.77 | 4.8 | 0.74 | 1.9 | 0.24 | 3.9 | 0.24 | 2.1 | 0.43 |
| CB-180 | n.d. |  | n.d. |  | n.d. |  | n.d. |  | 3.6 | 0.88 | 1.0 | 0.15 |
| ∑PCBs | 0.81 |  | 15 |  | 11 |  | 11 |  | 14 |  | 10 |  |

Table S4. Concentrations (ng/g fat) of individual PBDE congeners and HBCDDs in *Mytilus edulis* (ME), *Cyclina sinensis* (CS)*, Ruditapes philippinarum* (RP) *and Sinonovacula constricta* (SC) from Weihai (WH), Zhoushan (ZS) and Nantong (NT), ZS1 and ZS2 were natural and cultivated mussel, respectively.

| Compound | YS (ME)  (n=3) | | ZS1 (ME)  (n=3) | | ZS2 (ME)  (n=3) | | NT (CS)  (n=3) | | NT (RP)  (n=3) | | NT (SC)  (n=3) | |
| --- | --- | --- | --- | --- | --- | --- | --- | --- | --- | --- | --- | --- |
|  | mean | S.D. | mean | S.D. | mean | S.D. | mean | S.D. | mean | S.D. | mean | S.D. |
| *Lipid (%)* | 2.6 | 0.11 | 1.6 | 0.19 | 2.0 | 0.27 | 1.6 | 0.050 | 0.97 | 0.030 | 2.2 | 0.090 |
| BDE-47 | 3.3 | 0.54 | 2.0 | 0.24 | 1.3 | 0.24 | 1.6 | 0.50 | 1.6 | 0.22 | 1.9 | 0.46 |
| BDE-85 | 1.1 | 0.20 | n.d. |  | n.d. |  | n.d. |  | n.d. |  | n.d. |  |
| BDE-99 | 0.56 | 0.13 | 0.74 | 0.15 | 1.20 | 1.1 | 0.92 | 0.12 | <LOQ (2)^a^ |  | 0.57 | 0.070 |
| BDE-100 | 1.2 | 0.15 | 0.41 | 0.020 | 0.38 | 0.21 | 0.21 | 0.060 | 0.30 | 0.040 | 0.19 | 0.040 |
| BDE-153 | 0.22 | 0.15 | 0.36 | 0.080 | 0.44 | 0.12 | 0.19 | 0.010 | 0.23 | 0.020 | 0.19 | 0.010 |
| BDE-154 | 1.5 | 0.30 | 1.0 | 0.19 | 0.85 | 0.26 | 1.0 | 0.16 | 0.94 | 0.15 | 1.1 | 0.040 |
| BDE-183 | 0.51 | 0.010 | <LOQ(3)^a^ |  | <LOQ (3)^a^ |  | <LOQ (3)^a^ |  | <LOQ (3)^a^ |  | 0.56 | 0.020 |
| ∑PBDEs^a^ | 8.4 |  | 4.6 |  | 4.1 |  | 4.2 |  | 3.5 |  | 4.5 |  |
| HBCDD | 40 | 7.8 | 32 | 7.1 | 21 | 8.3 | 42 | 8.1 | 34 | 4.5 | 38 | 0.34 |

^a^ Below limit of quantification (LOQ), number of samples below LOQ are given in parentheses

Table S5. Limit of quantification (ng/g fat) for PBDEs in *Mytilus edulis* (ME), *Cyclina sinensis* (CS)*, Ruditapes philippinarum* (RP) *and Sinonovacula constricta* (SC) from Weihai (WH), Zhoushan (ZS) and Nantong (NT), ZS1 and ZS2 were natural and cultivated mussel, respectively.

|  | BDE-47 | BDE-85 | BDE-99 | BDE-100 | BDE-153 | BDE-154 | BDE-183 |
| --- | --- | --- | --- | --- | --- | --- | --- |
| YS (ME) | < 0.37 | < 0.03 | < 0.30 | < 0.07 | < 0.06 | < 0.06 | < 0.30 |
| ZS1(ME) | < 0.51 | < 0.05 | < 0.42 | < 0.10 | < 0.08 | < 0.08 | < 0.42 |
| ZS2(ME) | < 0.61 | < 0.06 | < 0.50 | < 0.12 | < 0.09 | < 0.10 | < 0.50 |
| NT (CS) | < 0.66 | < 0.06 | < 0.53 | < 0.13 | < 0.10 | < 0.11 | < 0.53 |
| NT (RP) | < 1.06 | < 0.10 | < 0.86 | < 0.22 | < 0.16 | < 0.17 | < 0.86 |
| NT (SC) | < 0.47 | < 0.04 | < 0.38 | < 0.10 | < 0.07 | < 0.08 | < 0.38 |

Table S6. Concentrations (ng/g fat) of individual MeO-PBDE congeners in *Mytilus edulis* (ME), *Cyclina sinensis* (CS)*, Ruditapes philippinarum* (RP) *and Sinonovacula constricta* (SC) from Weihai (WH), Zhoushan (ZS) and Nantong (NT), ZS1 and ZS2 were natural and cultivated mussel, respectively.

| Compound | WH (ME)  (n=3) | | ZS1 (ME)  (n=3) | | ZS2 (ME)  (n=3) | | NT (CS)  (n=3) | | NT (RP)  (n=3) | | NT (SC)  (n=3) | |
| --- | --- | --- | --- | --- | --- | --- | --- | --- | --- | --- | --- | --- |
|  | mean | S.D. | mean | S.D. | mean | S.D. | mean | S.D. | mean | S.D. | mean | S.D. |
| *Lipid (%)* | 2.6 | 0.11 | 1.6 | 0.19 | 2.0 | 0.27 | 1.6 | 0.050 | 0.97 | 0.030 | 2.2 | 0.090 |
| 6-MeO-BDE-47 | 22 | 2.1 | 8.2 | 2.4 | 5.5 | 1.8 | 1.9 | 0.65 | 1.3 | 0.35 | 4.6 | 0.41 |
| 2’-MeO-BDE-68 | 14 | 1.6 | 4.2 | 1.3 | 2.4 | 1.0 | 0.96 | 0.32 | 1.3 | 0.34 | 2.1 | 0.17 |
| 6-MeO-BDE-85 | n.d. |  | n.d. |  | n.d. |  | n.d. |  | n.d. |  | n.d. |  |
| 6-MeO-BDE-90 | 0.34 | 0.020 | n.d. |  | n.d. |  | n.d. |  | n.d. |  | n.d. |  |
| 6-MeO-BDE-99 | 0.52 | 0.020 | 0.073 | 0.017 | 0.099 | 0.064 | n.d. |  | n.d. |  | n.d. |  |
| 2-MeO-BDE-123 | n.d. |  | n.d. |  | n.d. |  | 0.23 | 0.024 | 0.22 | 0.024 | 0.31 | 0.019 |
| 6-MeO-BDE-137 | 4.5 | 0.71 | n.d. |  | n.d. |  | n.d. |  | n.d. |  | n.d. |  |
| ∑MeO-PBDEs | 41 |  | 12 |  | 8.1 |  | 3.1 |  | 2.9 |  | 7.1 |  |
